# 78. Atypical presentation of anti-signal recognition particle antibody positive myositis with profound extra-muscular features

**DOI:** 10.1093/rap/rky034.041

**Published:** 2018-09-20

**Authors:** Abeer Ghuman, Richard Stratton

**Affiliations:** Rheumatology, Royal Free Hospital, London, UNITED KINGDOM

**Introduction:** Autoantibodies against signal recognition particle (SRP) were first reported in the serum of a patient with polymyositis. Anti-SRP antibodies are a rare cause of myopathy; they are found in roughly 5% of all inflammatory myopathies.Based on clinical observations, it is reported that anti-SRP antibodies are associated with the severe and rapidly progressive muscle weakness, marked elevation of creatine kinase (CK) and a necrotising myopathy on histology. Extramuscular manifestations are commonly low in incidence. Patients with anti-SRP positive myositis tend to show a good initial response to high dose steroid therapy but the myositis then tends to relapse leading to the requirement of high cumulative doses of steroid. As well as steroid therapy these patients do commonly require additional immunotherapy. No particular immunosuppressive agent or treatment regime has been found to be consistently effective or superior in these patients. We describe a case of anti-SRP positive myositis presenting with profound extramuscular features including lung and cardiac involvement in the absence of limb, trunk or bulbar weakness. This patient showed a marked clinical response to high dose steroids and early B cell depletion with rituximab.

**Case description:** A 63 year old Malaysian gentleman developed an acute onset of a pruritic rash over his back. The rash was erythematous, flat and confluent and spread at sites where he scratched. The rash continued to progress despite topical corticosteroids. He was given a seven day course of prednisolone from his general practitioner (GP) which settled the rash. As the rash subsided he developed acute onset of Raynauds symptoms. He then developed rapidly progressive shortness of breath over the course of three months. His exercise tolerance became limited to a few hundred yards. There was no associated cough or chest pain. Associated with this shortness of breath was severe fatigue. He developed sweats and fevers at night. There was no history of limb weakness or dysphagia. He has no significant past medical history and was on no regular medications. He is a lifelong non-smoker. He reattended his GP surgery who suspected an autoimmune aetiology for his symptoms and sent an autoimmune screen. The anti-nuclear antibody (ANA) returned strongly positive and he was referred on to rheumatology clinic. On arrival in rheumatology clinic three months after onset of respiratory symptoms he was markedly hypoxic with saturations of 90% on air and he was tachypnoeic with a respiratory rate of 30. He was tachycardic at 100 beats per minute. He spiked fevers of over 39 degrees celsius typically at night during his inpatient stay. He had a mild synovitis affecting his metacarpophalangeal and proximal interphalangeal joints bilaterally. There was no visible rash. Chest examination revealed bibasal fine inspiratory crackles. There was no focal neurology and he had a normal medical research council (MRC) muscle score at presentation. Bloods showed raised inflammatory markers with an erythrocyte sedimentation rate (ESR) 103mm/hr and C-reactive protein 36mg/ml. CK was high at 1083unit/L and troponin T 368ng/L. Renal function was normal and urine dip was negative for blood and protein. Autoimmune screen revealed positive ANA 1:5120 nucleolar, negative extranuclear antigen (ENA), normal complement and normal double stranded DNA. The myositis panel came back as positive for anti-SRP antibodies He had a chest x-ray which showed bilateral pulmonary infiltrates. High resolution computed tomography (CT) chest showed areas of interstitial thickening and atelectasis within the middle lobe, lingula and the lower lobes bilaterally with scattered areas in the apices. There was traction dilatation of the distal airways involving both lower lobes and shallow bilateral effusions. There was no lymphadenopathy. The imaging was discussed at a respiratory multidisciplinary meeting, the findings suggested a fibrotic process which were not entirely typical of either non specific interstitial pneumonia (NSIP) or usual interstitial pneumonia (UIP). Lung function showed preserved forced expiratory volume (FEV1) and forced vital capacity (FVC) but a low transfer factor (DLCO). Electrocardiogram (ECG) showed sinus tachycardia. Due to the raised troponin he went on to have a cardiac magnetic resonance imaging (MRI) which showed late gadolinium enhancement at the level of the superior left ventricle/right ventricle insertion point which minimally extend into the septum (non ischaemic distribution). The finding on T1 and T2 suggested an early stage of cardiac involvement of connective tissue disorder. An electromyogram (EMG) showed focal myopathic features only in the right iliapsoas muscle with evidence of some muscle membrane instability. There was no associated clinical evidence of weakness in this muscle distribution. Nail fold capillaroscopy demonstrated abnormal capillaries with dropout from the rows. The diagnosis of myositis with lung, cardiac and joint involvement was made. He was treated with three days of intravenous (IV) methylprednisolone and then 40mg oral prednisolone once daily. He then had Rituximab infusion at a dose of 1g two weeks apart. He was then started on mycophenalate mofetil at 500mg BD as maintenance therapy. He showed a dramatic improvement with treatment - he was no longer oxygen dependent after three days of intravenous steroid and was saturating at 98% on air. His synovitis resolved. His ESR and CRP reduced to normal. His CK level came down to 446unit/L. He is currently being followed up as an outpatient and remains on low dose oral steroid and mycophenalate mofetil.

**Discussion:** Anti-SRP antibodies are typically considered serological markers of severe necrotising myopathy. The largest retrospective case study of one hundred anti-SRP positive patients reported that all the patients presented with weakness and reported low incidence of intramuscular manifestations. This case is an atypical presentation of this disease because he presents with prominent rapidly progressive extramuscular features with only mild muscle involvement clinically and on biochemistry and EMG. As he had developed cardiac involvement and interstitial lung disease with associated progressive symptoms rapidly over a matter of months he needed urgent and aggressive treatment. There is no reported consensus on a regimen for immunosuppression in these cases due to the rarity of the disease. With any form of myositis expert opinion suggests use of high dose steroid followed by maintenance immunosuppression. Anti-SRP positive patients were well represented in the largest randomised controlled trial of rituximab in myositis which showed it to be beneficial in the disease with a significant steroid sparing effect. However there are case reports of poor response of these patients to rituximab. These patients tend to relapse and so need close follow up during their treatment and may require additional immunosuppression and large cumulative doses of steroid.

**Key Learning Points:** Anti-SRP positive myositis can present atypically with profound extramuscular features which may delay diagnosis, it is important to be aware of this as they require early and aggressive treatment. As anti-SRP positive myositis is rare it is difficult to get a consensus on the most effective immunosuppressant regime for these patients. Early initiation of B cell depletion with rituximab has shown positive results in the management of extramuscular manifestations in an anti-SRP positive patient.

**Disclosure: A. Ghuman:** None. **R. Stratton:** None.

